# A microfluidic platform for the synthesis of polymer and polymer-protein-based protocells

**DOI:** 10.1140/epje/s10189-024-00428-5

**Published:** 2024-06-03

**Authors:** Jessica Ann O’Callaghan, Neha P. Kamat, Kevin B. Vargo, Rajarshi Chattaraj, Daeyeon Lee, Daniel A. Hammer

**Affiliations:** 1 Department of Chemical and Biomolecular Engineering, University of Pennsylvania, 210 S 33rd Street, Philadelphia, PA 19104 USA; 2 Department of Biongineering, University of Pennsylvania, 210 S 33rd Street, Philadelphia, PA 19104 USA

## Abstract

**Abstract:**

In this study, we demonstrate the fabrication of polymersomes, protein-blended polymersomes, and polymeric microcapsules using droplet microfluidics. Polymersomes with uniform, single bilayers and controlled diameters are assembled from water-in-oil-in-water double-emulsion droplets. This technique relies on adjusting the interfacial energies of the droplet to completely separate the polymer-stabilized inner core from the oil shell. Protein-blended polymersomes are prepared by dissolving protein in the inner and outer phases of polymer-stabilized droplets. Cell-sized polymeric microcapsules are assembled by size reduction in the inner core through osmosis followed by evaporation of the middle phase. All methods are developed and validated using the same glass-capillary microfluidic apparatus. This integrative approach not only demonstrates the versatility of our setup, but also holds significant promise for standardizing and customizing the production of polymer-based artificial cells.

**Graphical Abstract:**

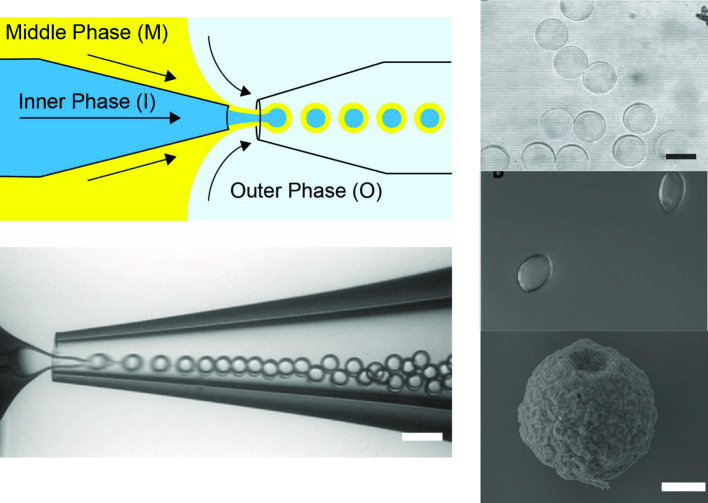

**Supplementary Information:**

The online version contains supplementary material available at 10.1140/epje/s10189-024-00428-5.

## Introduction

Over the past two decades, fabrication of artificial biomimetic materials, including artificial cells, or protocells, has attracted significant attention [1–4]. Interest in the assembly of artificial cells was at first motivated by an attempt to recapitulate cell structure and function, but has since expanded to include specific applications like environmental remediation [5] and biotechnology [6, 7]. One way to design protocells is to use a bottom-up approach, which involves assembling the minimal components of a cell from scratch [8]. In its simplest form, a protocell has a compartmentalized layer, modeled after the cell’s bilayer membrane [9]. Vesicles with bilayers membranes are often considered suitable protocell scaffolds. Initially, lipid-based vesicles, or liposomes, dominated early efforts to recreate essential cellular processes [9]; these efforts were reasonable since lipids are the principal component of most biological cell membranes. However, vesicles built from block copolymers, called polymersomes, offer significant advantages, including higher stretching elasticity and consequently greater resistance to lysis, as well as the potential for additional functionalization through polymer chemistry [10–13]. Notably, these advantages are not limited to polymersomes, but rather extend to other polymer-based systems like microcapsules, which are composed of an inner aqueous core surrounded by a polymeric shell [14, 15]. With this perspective in mind, we focus on the development of polymer-based protocells, as we believe their boundaries possess superior and tunable properties, which can facilitate their use in a variety of fields.

Conventional methods for preparing polymer-based protocells, like thin-film rehydration [11, 16, 17] or electroformation [10] used in polymersome assembly, are often plagued by significant batch-to-batch variability, leading to poor control over their properties [18, 19]. As such, a major challenge in the field of protocell engineering has been the development of high-throughput, easily operable, and controllable methods [4]. In recent years, microfluidics has drawn increasing attention as an efficient and reproducible route for generating uniform emulsion droplets, which can be equipped with building block components for protocell assembly [20]. Several microfluidic methods for preparing artificial cells have already been described, including by Weitz and coworkers, who used a capillary microfluidic device to produce unilamellar liposomal vesicles from droplets with ultrathin oil shells [21]. Recently, Petit and coworkers developed a PDMS-based microfluidic platform for preparing liposomes and polymersomes [22]. Ordered structures that are not vesicles have also been developed, including by van Hest and coworkers, who harnessed coacervate self-assembly in microfluidic-generated droplets [23]. Notably, Raghavan and coworkers fabricated biopolymer microcapsules with compartmentalized architecture using an oil-free water-gas microfluidic technique [24].

These studies collectively demonstrate the potential for microfluidic technology to advance protocell development. With the advantages of polymers in mind, and recognizing the capability of microfluidic technology to accelerate fabrication of artificial cells with diverse functions and features, additional strategies must be explored. In this study, we introduce novel techniques for preparing protocells based on three distinct polymer morphologies - polymersomes, protein-integrated polymersomes, and polymeric microcapsules - using the same glass-capillary microfluidic setup. The versatility of the device stems from its compatibility with multiple organic solvents, selected based on different polymer solubilities. We demonstrate that by manipulating the compositions of the droplet phases and the assembly flow rates, we can reliably tune the properties of each protocell type. Since multiple scaffolds can be produced using the same equipment, each offering unique benefits for specific applications, our findings aim to help streamline the production of artificial cells.

## Materials and methods

### Fabrication of capillary microfluidic devices

The microfluidic devices are based on coaxial assemblies of round and square glass capillaries developed by Weitz and coworkers [25, 26]. In brief, two round capillaries of outer diameter 1.03–1.05 mm (World Precision Instrument Inc.) are precisely tapered to achieve orifice diameters of 20–40 $\upmu$m for the injection of the inner phase and 80–150 $\upmu$m for the collection of double emulsions using a microcapillary puller (P-1000, Sutter Instrument Inc.) and a microforge machine (Narishige MF-830). The outer surface of the inner capillary is treated with octadecyltrichlorosilane (OTS, ThermoFisher Scientific) to render it hydrophobic and improve the wettability of the middle phase, which flows around it in the same direction as the inner phase. The round capillaries are inserted into each end of the square capillary, which has the same inner dimension as the outer diameters of the round capillaries, ensuring good coaxial alignment, and sealed using 5 min Epoxy (Devcon).

### Polymersome fabrication

Polymersomes are prepared using poly(ethylene oxide-b-1,2-butadiene) (Polymer Source). Molecular weights for the ethylene oxide and butadiene blocks are 1.3 and 2.5, 2 and 3.8, and 4.8 and 5.2 kg mol^-1^, respectively. These polymers are designated as PEO_30_-b-PBD_46_, PEO_45_-b-PBD_70_, and PEO_109_-b-PBD_92_, where the subscript indicates the number of repeat units in each block. To prepare the polymersomes, these block copolymers are dissolved in a binary mixture of chloroform (CHCl_3_, Sigma-Aldrich) and hexane (ThermoFisher Scientific) at a concentration of 2 mg/mL. The volume ratios of chloroform to hexane (chlorform:hexane) for PEO_30_-b-PBD_46_, PEO_45_-b-PBD_70_, and PEO_48_-b-PBD_52_ are adjusted to 30:70, 33:67, and 38:62, respectively.

To generate double emulsions for polymersome assembly, we utilize fluid phases comprising 95 mg/mL sucrose (ThermoFisher Scientific), the aforementioned block copolymer solution, and 2 wt% poly(vinyl alcohol) (PVA, 87–89% hydrolyzed, M_w_ = 13,000–23,000 g mol^-1^, Sigma-Aldrich) in PBS. Additionally, 0.5 wt% Pluronic F-68 (ThermoFisher Scientific) is dissolved in the PBS solution. These components are introduced into the microfluidic device as the inner (I), middle (M), and outer (O) phases, respectively, and are controlled by three syringe pumps (PHD, Harvard Apparatus) at flow rates of 1, 4–6, and 15–30 mL/hr. Specific flow rates for each experiment are detailed in Fig. 2d. To visualize as-formed polymersomes, fluorescein isothiocyanate-dextran (FITC-dextran, MW= 10 kg mol^-1^, Sigma-Aldrich) is added to the inner phase at a concentration of 1 mg/mL and Nile Red (Sigma-Aldrich) is added to the middle phase at a concentration of 20 $\upmu$g/mL. To explore the role of F-68 in the dewetting process, emulsions are also prepared using 2 wt% PVA as the outer phase, without F-68. The emulsions are collected in a semienclosed silicone isolation chamber (diameter 9 mm, height 0.12mm, SecureSeal) and covered with a glass coverslide for further characterization. The dewetting process and resultant polymersomes are observed using upright (Carl Zeiss Axio Plan II) and confocal (Leica DMi8) microscopes.

### Protein-blended polymersome fabrication

Protein-blended polymer vesicles are constructed using droplets stabilized by PEO_30_-b-PBD_46_, with engineered recombinant proteins in various phases. A key protein used is oleosin, a naturally-occurring surfactant found in sunflower seeds, known for stabilizing oil bodies. Oleosin is an amphiphilic protein with the sequence structure X-H-Y, where X and Y are the hydrophilic N- and C-terminal arms, and H is the hydrophobic block. Our laboratory previously modified oleosin’s structure by truncating all 3 components, allowing it to assemble into different suprastructures like vesicles, micelles, sheets, and fibers [27, 28]. A notable variant is Oleosin 30 G, which has a shortened and more flexible hydrophobic core consisting of 30 residues, as well as additional glycine residues. This modification makes Oleosin 30 G slightly more water-soluble compared to longer hydrophobic versions, while retaining its amphiphilic nature. The X-H-Y sequence of 30 G is represented as 43–30 G-62. This protein by itself forms micelles with a critical micelle concentration (CMC) of 4.1 $\upmu$M, as previously described [28]. The plasmid is grown in E.Coli (BL21-DE3), and the protein is purified using immobilized metal affinity chromatography (IMAC) and dialyzed into 1x PBS. During polymersome assembly, oleosin is present at a concentration of 0.4 mg/ml in the inner phase, while the middle phase comprises 1 mg/mL PEO_30_-b-PBD_46_ in a mixture of toluene and chloroform (72:28 by volume). The flow rates of the inner, middle, and outer phases are set to 2000 $\upmu$L/hr, 7000 $\upmu$L/hr, and 20 mL/hr, respectively.

Next, we engineered a version of 30 G by further shortening the hydrophilic arms and introducing anionic groups, enhancing protein expression and solubility. This new variant, designated as 25–30 G-30(-), is introduced at a concentration of 0.2 mg/mL in the inner phase of PEO_30_-b-PBD_46_-stabilized droplets prepared via F-68 assisted dewetting. Biotin is attached to the N-terminus of 25–30 G(-)-30 using EZ-Link^TM^ Sulfo-NHS-LC-Biotin (ThermoFisher Scientific), made possible by the lysine-modified N-terminus of the protein, which reacts with the NHS group on biotin. The microfluidic assembly conditions for these vesicles are consistent with those used for PEO_30_-b-PBD_46_ vesicles. After assembly, the polymersomes are incubated with Avidin (Alexa Fluor^TM^ 488 conjugate, ThermoFisher Scientific) for 15–30 min, and then imaged using fluorescence microscopy on an inverted epifluorescent microscope (Nikon Eclipse TE300).

In the third experiment, recombinant eGFP, made as previously described [29], is incorporated at a concentration of 1 mg/ml in the outer phase during microfluidic assembly. Vesicles are assembled and annealed by solvent evaporation over one week. After assembly, the vesicles are washed to removal any residual eGFP from the outer phase, and then imaged by fluorescence microscopy on an inverted epifluorescent microscope.

### Microcapsule fabrication

Microcapsules are prepared using acid-terminated poly(D,L-lactic-co-glycolic acid) (PLGA) with a composition of 75:25 (0.84 dL g-1 in CHCl_3_, Lactel). The double-emulsion templates used for microcapsule assembly are prepared with an inner phase composed of 25 mM NaCl dissolved in DI water. The middle fluid consists of 30 mg/mL PLGA dissolved in CHCl_3_, with the addition of either 0.15, 0.25, or 0.4 wt% hydrophobic silica nanoparticles dissolved in toluene (Nissan Chemical Inc.). These varying nanoparticle concentrations are used to create microcapsules with smooth, rough, or very rough textures, respectively. Nile Red is also added to the middle phase for fluorescent visualization of the shell. The outer phase of the emulsion consists of 1 wt% PVA.

The double emulsions are collected in petri dishes containing 0.1, 0.25, 0.5, or 1 M NaCl to osmotically anneal the microcapsules and tune their diameters. After collection, petri dishes are covered with aluminum foil to minimize CHCl_3_ evaporation. After 2 hrs, when the droplets reach their equilibrium size, the aluminum foil is removed to allow for evaporation of CHCl_3_ for 48 hrs, leaving behind a solidified PLGA shell. The average outer diameter (D) of dried microcapsules is measured by taking mid-plane images on a confocal microscope (Leica Stellaris 5, 63x magnification, mid-plane imaging, N $\ge$ 200 microcapsules/type). To characterize differences in surface roughness at different nanoparticle concentrations, the capsules are imaged by scanning electron microscopy (SEM) (JEOL JSM-7500F).

## Results

### Polymersome formation

Polymersomes are prepared via a surfactant-assisted dewetting process from water-in-oil-in-water (W/O/W) double emulsions. Inspired by the work of Deng and coworkers, this platform relies on careful manipulation of the interfacial energies of block copolymer-stabilized drops using a triblock copolymer surfactant, Pluronic F-68 [30].

The key to dewetting double emulsions lies in the spreading coefficient of each phase (Eq. 1), which is defined as:

1 $$\begin{aligned} S_i = \gamma _{jk} - (\gamma _{ij} + \gamma _{ik}) \end{aligned}$$

where $\gamma _{jk}$ is the interfacial tension between phases *j* and *k* [31]. When the spreading coefficients for all three phases are negative, polymer-stabilized drops tend to adopt an acorn-like equilibrium structure. In this case, the surface energy between the inner core and oil shell phase is comparable to that between the inner core and the external phase. Several research groups have observed partial dewetting configurations for block copolymer-stabilized drops, which ultimately form polymersomes whose membranes have a patch of excess polymer where the organic solvent drop was attached [32–35].

**Fig. 1:**
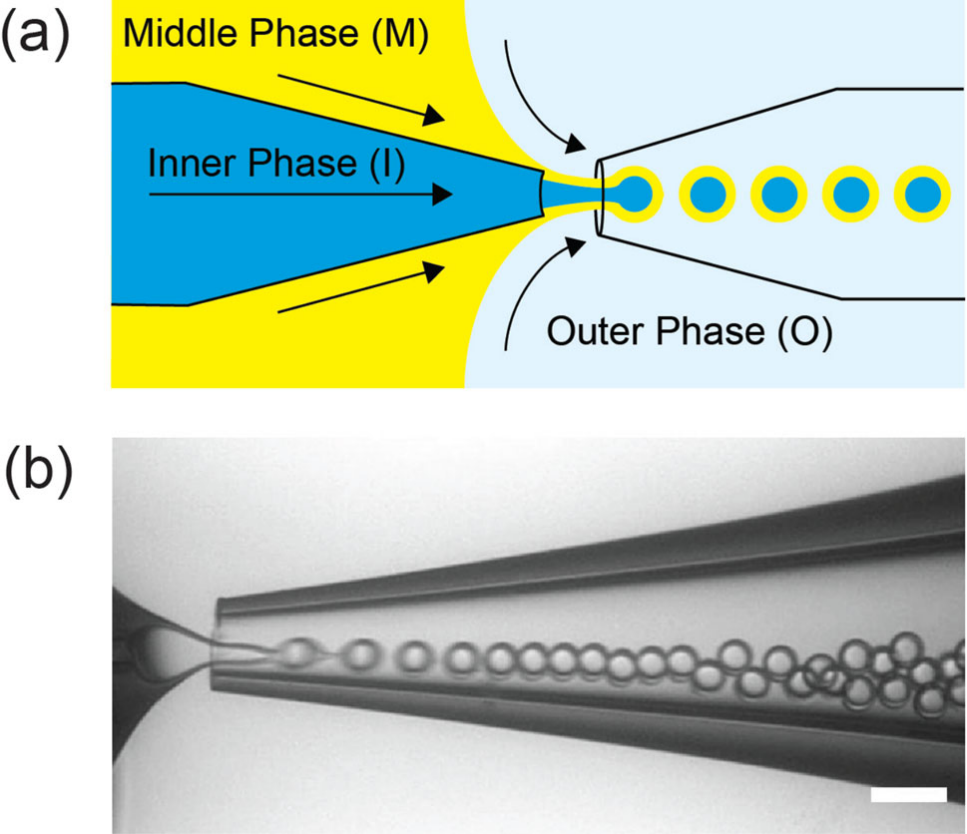
**a** Schematic and **b** micrograph of double-emulsion production using a glass-capillary microfluidic device (scale bar = 50 $\upmu$m)

**Fig. 2:**
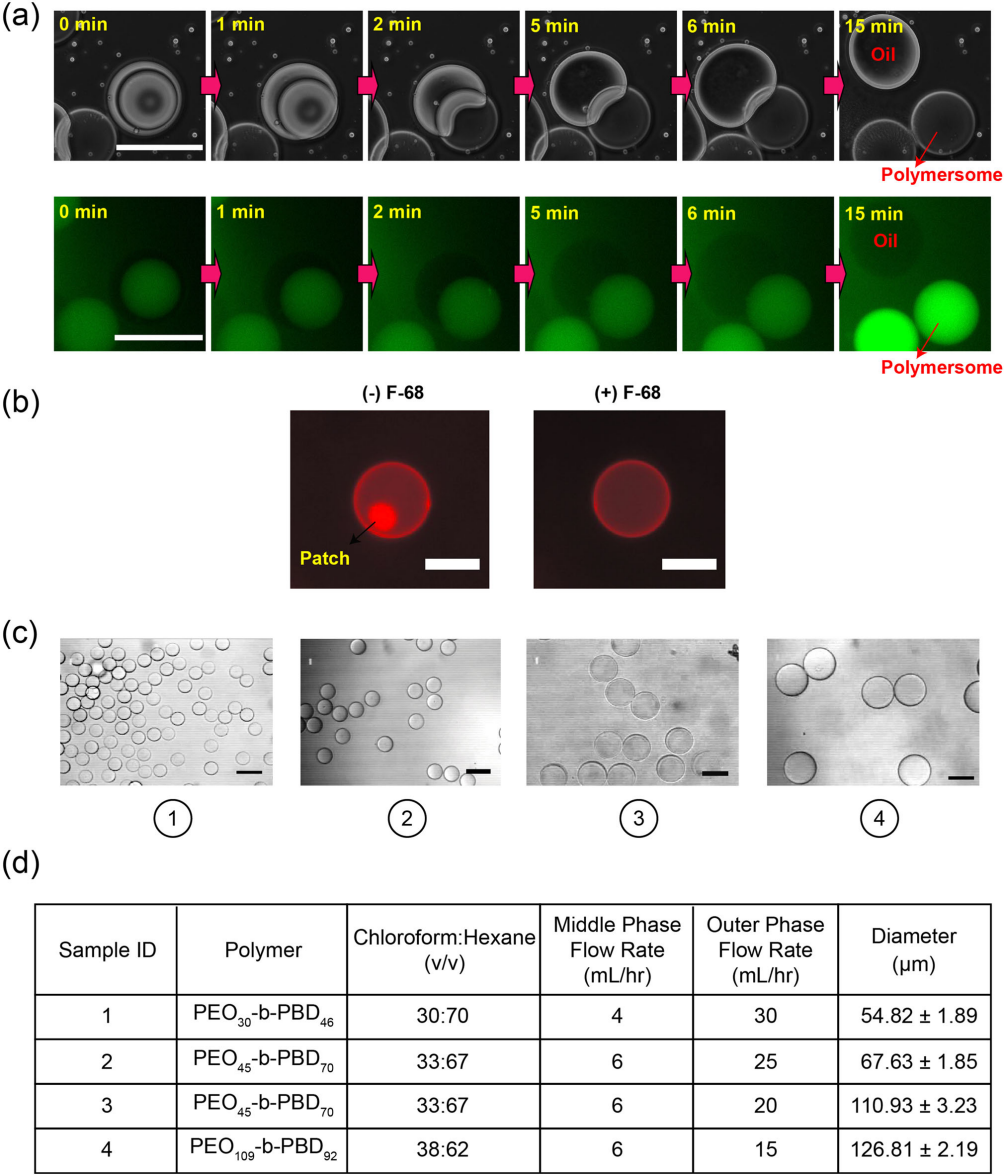
**a** Optical (top) and fluorescent (bottom) time-series of complete dewetting of polymersomes separating from oil droplets (scale bar = 100 $\upmu$m). Fluorescent polymersomes contain FITC-dextran inside. **b** Fluorescent images of bilayer membranes (red) prepared with and without F-68 (scale bar = 50 $\upmu$m). **c** Optical images of polymersomes prepared by surfactant-assisted dewetting (scale bar = 100 $\upmu$m) and (d) associated experimental conditions, resulting in a wide range of polymersome diameters (n = 50 polymersomes/type, mean ± std. dev)

Slow evaporation of the middle phase, as well as reported destabilizing effects of the polymer patch [32], has limited the utilization of polymersomes prepared by microfluidics. Therefore, we are interested in preparing polymersomes with homogeneous membranes by complete dewetting of the oil phase from the inner core. This requires a high interfacial tension between the core and shell, where $S_{O}>0$. Deng and coworkers showed that Pluronic F-68 can be used to adjust the interfacial energies of a lipid-stabilized droplet to satisfy this requirement, ensuring complete dewetting and formation of oil-free liposomes [30]. To investigate whether Pluronic F-68 can similarly induce complete dewetting in polymer-stabilized droplets, we first prepare double-emulsion drops in a capillary microfluidic device (Fig. 1). The inner (I), middle (M), and outer (O) phases comprise a sucrose solution, poly(ethylene oxide-b-1,2-butadiene) in a mixture of chloroform and hexane, and PVA with F-68 in PBS, respectively (see Fig. 2d for more experimental details). Chloroform dissolves PEO-b-PBD in the middle phase, while hexane provides sufficient surface tension between the oil and aqueous phases for droplet formation to occur. The ratio of chloroform to hexane selected for each block copolymer sample provides a suitable combination of these properties. Freshly prepared emulsions are collected in a sealed observation chamber and observed immediately. To monitor the dewetting process, fluorescein isothiocyanate-dextran (FITC-dextran) is added to the inner phase in some experiments. As shown in Fig. 2a, at 0.5 wt% F-68, the dewetting process is recorded using optical and fluorescence microscopy; complete separation of the inner droplet from the oil shell occurs within 15 min (Videos S1 and S2). This results in the formation of polymersomes with uniform membranes and oil droplets with excess polymer (Fig. 2b, right). In contrast, in the absence of F-68, polymersomes with patches of excess polymer are observed (Fig. 2b, left).

To assess whether the mechanisms for F-68-assisted dewetting are similar to those described for lipid-stabilized droplets, we conduct interfacial tension measurements using the pendant drop method (see Supplementary Information for details), with PEO_30_-b-PBD_46_ in the oil phase. However, maintaining the drop on the dispensing needle in the presence of F-68 presents challenges, complicating the acquisition of reliable equilibrium surface tension readings. When examining the interfacial tension between the middle and outer (M-O) phases, $\gamma _{\textrm{MO}}$, with and without F-68, at the moment when the oil droplet typically detaches in the presence of F-68 ($\approx$ 1 min), $\gamma _{\textrm{MO}}$ is observed to be lower in the presence of F-68 (see Figure S1 in Supplementary Information). This observation suggests that F-68 adsorption to the external water-oil interface leads to a reduction in $\gamma _{\textrm{MO}}$. Moreover, adhesion between droplets of the inner and outer phases, which is performed to estimate the tension between them (see Supplementary Information for details) is exclusively observed when F-68 is present in the outer phase, indicating F-68 reduces the interfacial tension between the two surfactant monolayers. This interplay of interfacial tensions in the presence of F-68 appears to facilitate the expulsion of the oil shell, thereby promoting the formation of an intact bilayer membrane [30].

Surfactant-assisted dewetting is accomplished with various block copolymers dissolved in different solvent ratios of chloroform and hexane (Fig. 2c and d). By carefully adjusting the flow rates of the middle and outer phases, we can manipulate the diameters of the polymersomes, ranging from 55 to 127 $\upmu$m. We observe that polymersomes made with PEO_30_-b-PBD_46_ and PEO_45_-b-PBD_70_ both dewet within 15 min, whereas polymersomes made with PEO_109_-b-PBD_92_ dewet in $\approx$ 3 h. One possible explanation for this stems from differences in the hydrophilic fractions of the copolymers. Specifically, adsorption of F-68 may be greater when the block copolymer hydrophilic fraction is lower. As chloroform evaporates, leading to a reduction in solvent quality, these polymers are more likely to remain dissolved in the middle phase. Consequently, this may lead to a quicker reduction in interfacial energy, as more F-68 is able to crowd at the interface, accelerating the dewetting process. However, since multiple experimental parameters are changed for different PEO-b-PBD samples, additional experiments are needed in order to test these hypotheses.

### Protein-blended polymersome formation

**Fig. 3:**
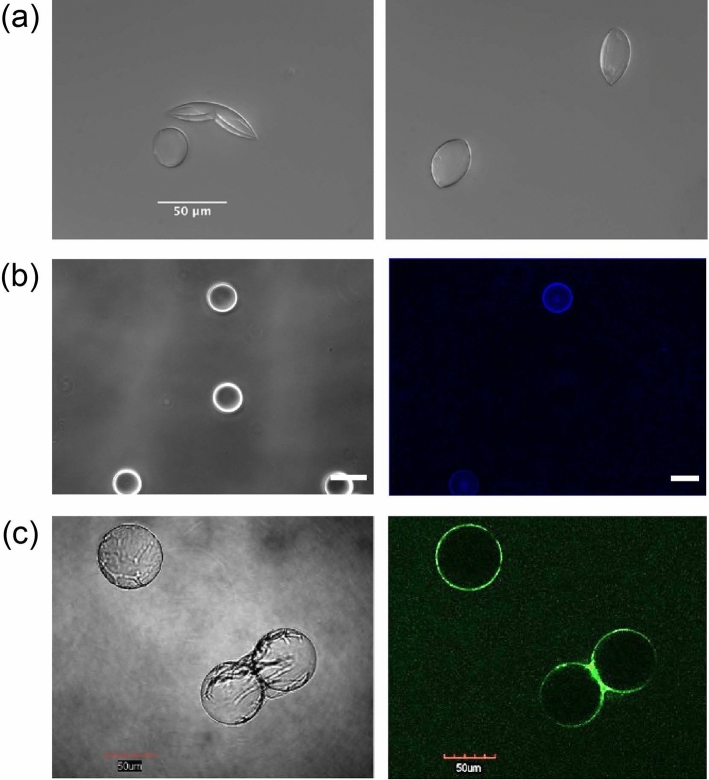
**a** Incorporation of oleosin 30 G in the inner phase of polymersomes made of PEO_30_-b-PBD_46_. **b** Bright-field and fluorescent images of avidin (Alexa Fluor^TM^ 488 conjugate) incubated with PEO_30_-b-PBD_46_ polymersomes prepared with biotin-functionalized 25–30 G-30(-) and F-68 in the inner and outer phases, respectively. **c** Incorporation of eGFP in the outer phase of polymersomes made of PEO_30_-b-PBD_46_

As described in the methods, we incorporate three distinct proteins into PEO_30_-b-PBD_46_ membranes during microfluidic assembly. First, we incorporate a variant of oleosin, 43–30 G-62 (also referred to as oleosin-30 G), into the inner phase of the droplet. Since the hydrophobic domains of the oleosin variants are heavily truncated, which eliminates much of the proteins’ secondary structure and imparts chain-like surfactant behavior, we do not expect protein functionality to be compromised after membrane incorporation. The protein is seen to strongly associates with the polymersome inner leaflet, inducing a lensing and curvature to the polymersome (Fig. 3a). As an additional demonstration of protein functionality within the PEO-b-PBD membrane, we explore the surface accessibility of another variant, 25–30 G-30(-), an anionic version of oleosin-30 G. This protein, pre-functionalized with biotin, is added to the inner phase of PEO_30_-b-PBD_46_-stabilized droplets, prepared with F-68 in the outer phase. After emulsion and polymersome assembly, fluorescent avidin is added to the sample. The association of avidin to the vesicle surface, visible by fluorescence microscopy (Fig. 3b and S2), suggests that the oleosin variant has the desired orientation in the outer leaflet of the bilayer.

As a third demonstration of protein association with membranes, we incorporate a soluble, recombinant green fluorescent protein, eGFP, in the outer phase during assembly. The adsorption of eGFP to the external phase is clearly seen by fluorescence microscopy, signaling that the eGFP protein retains its conformational integrity after membrane incorporation (Fig. 3c, left). Had the protein’s confirmation been altered from incorporation, no fluorescent signal would be detected. The strong interaction between eGFP and the membrane induces vesicles to adhere by GFP cross-bridging (Fig. 3c, right). Therefore, our findings indicate that proteins with different sequences and charges can associate with the polymersome membrane, inducing structural changes in polymersomes and cross-bridging.

### Microcapsule formation

As an alternative to vesicle assembly, we can also use microfluidics to fabricate hollow poly(lactic-co-glycolic acid) PLGA microcapsules [36, 37]. Unlike polymersomes, uniform microcapsules are obtained by complete evaporation of the middle phase to form a solid polymer shell. One major advantage of microcapsule formation over polymersome formation is the ability to tune the microcapsule size. This is done by adjusting the salt concentration in the aqueous phases of the droplet, which triggers water transport across the oil shell, swelling or shrinking the inner core [37–40]. Our aim is to prepare microcapsules within the size range typical of biological cells ($\approx$ 10 to 50 $\upmu$m). Since PLGA microcapsules from $\approx$ 30 to 50 $\upmu$m in diameter have already been reported, our focus is on preparing microcapsules that are <30 $\upmu$m. To achieve this, we utilize a collection capillary with a diameter roughly half of the size typically used ($\approx$ 80 $\upmu$m). The reduction in size enables the generation of droplets with reduced lateral dimension, enabling the assembly of smaller microcapsules under the same tested osmotic pressure differences. Additionally, we strive to prepare microcapsules that possess the surface features of biological cells, which we achieve by adding hydrophobic silica (SiO_2_) nanoparticles to the middle organic phase, which adsorb to the droplet interfaces and alter its surface roughness upon drying [41].

**Fig. 4:**
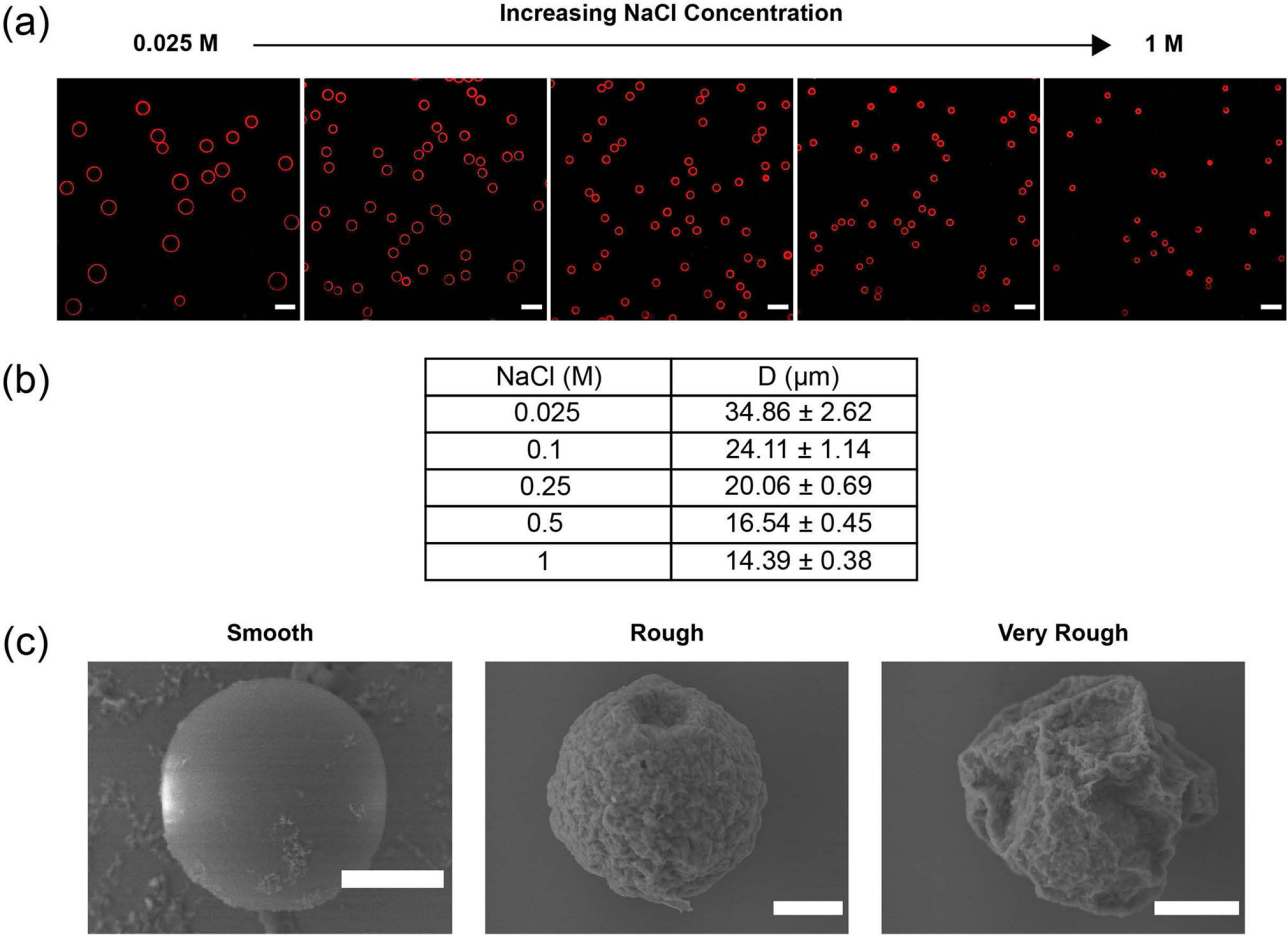
**a** Mid-plane confocal images of microcapsules with different sizes prepared in a single fabrication run (scale bar = 50 $\upmu$m).**b** Microcapsule diameters obtained from the addition of NaCl in the collection solution (N $\ge$ 200 microcapsules/type, mean ± std. dev.). **c** SEM micrographs of microcapsules prepared with a low (smooth), intermediate (rough), and high (very rough) concentration of SiO_2_ nanoparticles in the middle phase (scale bar = 5 $\upmu$m)

Microcapsules are first assembled from double-emulsion droplets whose inner, middle, and outer phases consist of a dilute NaCl solution, PLGA and SiO_2_ nanoparticles dissolved in chloroform, with the addition of Nile Red for fluorescent visualization of the shell, and PVA dissolved in water. The size of the microcapsules is adjusted by collecting the double emulsion into a NaCl solution with a different osmotic pressure than the internal phase. Since the starting inner droplet dimension is $\approx$ 35 $\upmu$m, we conduct all osmotic annealing experiments with hypertonic collection solutions, which leads to the extraction of water from the inner phase and volume reduction of the aqueous core. To delay solidification until the inner droplets reach the target size, double-emulsion samples are covered with tin foil. Once the droplet reaches its equilibrium size, which occurs in 2 h, the tin foil is removed and the middle phase is evaporated for 48 h. It is worth noting that, while not included in this study, microcapsules can be prepared with poly(ethylene-alt-maleic anhydride) (PEMA) in the outer phase instead of PVA. Stabilizers have been shown to integrate themselves into the surfaces of particles prepared from the emulsion templates they stabilize. As such, these microcapsules will possess a high surface density of carboxylic acid groups, making them suitable for ligand functionalization using carbodiimide chemistry [42, 43].

Microcapules ranging from 14 to 35 $\upmu$m in diameter are generated in a single fabrication run (Fig. 4a and b). As expected, the size of the microcapsule decreases as the concentration of salt in the collection solution increases. The smallest microcapsule size we observe ($\approx$ 14 $\upmu$m) is comparable to the size of a leukocyte, and, to the best of our knowledge, is the smallest PLGA microcapsule made by microfluidics reported to date. Furthermore, the surface roughness of PLGA microcapsules is tuned by controlling the concentration of SiO_2_ nanoparticles in the middle organic phase (Fig. 4c). When the SiO_2_ concentration is low, evaporation of chloroform leads to condensation of the dilute packing of nanoparticles at the external interface, leading to assembly of microcapsules with smooth surfaces. When the concentration is sufficiently high, the nanoparticles form a dense packing on the interface, which buckles as the solvent is removed, producing folds and crevices. Interestingly, the addition of more nanoparticles to the middle phase results in even larger features on the microcapsule surface.

## Discussion

The creation of artificial cells has huge implications for understanding cellular life and developing novel functions [2]. Microfluidics has the capacity to create artificial cells with accurate control over size, morphology, and composition, surpassing the limitations often encountered with traditional approaches [20]. To widen the range of properties and functionalities that artificial cells provide, we develop several new methods for fabricating polymer-based protocells, including polymersomes, protein-blended polymersomes, and polymeric microcapsule. These methods are all implemented on a common microfluidic device, helping provide a potential pathway toward streamlined polymer-based protocell scaffolding.

In our first experiment, we successfully fabricate uniform, single bilayer polymersomes using a dewetting strategy based on Pluronic F-68. To our knowledge, this represents the fastest microfluidic approach for producing uniform polymer vesicles, a significant advantage for limiting exposure of encapsulates to organic solvents in the emulsion. We observed that fully dewetted polymersomes prepared with various PEO-b-PBD samples display different dimensions, and, presumably, membrane permeabilities. For instance, the PBD block of PEO_109_-b-PBD_92_ has a molecular weight above its entanglement molecular weight [11], suggesting lower permeability than PEO_30_-b-PBD_46_ or PEO_45_-b-PBD_70_. It is worth acknowledging that polymersomes prepared with F-68 are more susceptible to degradation than those prepared without it, especially those that rapid dewet (within 48 h of assembly if <10 min). This suggests that the surfactant may alter the mechanical properties of the vesicle membrane in a way that reduces its stability, perhaps by reducing the area expansion modulus of the membrane, making it more flexible and less able to resist deformation [44]. Future enhancements in membrane stability could involve using high molecular weight polymers, which impart greater rigidity, albeit at the expense of a prolonged dewetting process. Additionally, assessing the differences in the area expansion modulus of polymer membranes prepared with and without F-68 using micropipette aspiration could provide valuable insights into the impact it has on the mechanical properties of the membrane.

Next, we explore integrating proteins into polymersome membranes. This involved two different charged proteins, oleosin-30 G and eGFP, which strongly associate with the polymersome membranes during assembly, and induce shape changes (oleosin-30 G) and adhesion (eGFP). Additionally, we incorporate biotin-functionalized 25–30 G(-)-30(-) into the membranes of polymersomes prepared by F-68-assisted dewetting. The association of avidin with the membrane suggests that these proteins are accessible on the surface of the polymersome, which would imply their integration into the outer leaflet during membrane assembly. However, these experiments only suggest biotin-avidin binding and call for more experiments, specifically incorporating a variant of the protein without biotin, to confirm that avidin’s recruitment to the surface is indeed mediated by biotin-avidin interactions. Successful demonstration of this recruitment could have significant implications for targeted delivery and biosensing applications, where interactions with specific biomolecules are crucial. This opens a promising avenue for future research, particularly in understanding the relationship between protein sequences and their interactions with polymersome membranes.

Lastly, we report on the assembly of cell-sized polymeric microcapsules with interesting surface features that resemble those found on natural cells. Microcapsules made from PLGA, an FDA-approved polymer, are already in clinical use for various applications [38, 39]. The ability to fabricate smaller microcapsules than those currently available broadens their potential biomedical applications, particularly where size is a limiting factor.

In summary, this study introduces new, efficient methods for artificial cell fabrication, all facilitated by a single microfluidic device. Once device fabrication is mastered, albeit challenging, assembly processes can be harmonized, allowing for versatile protocell scaffolding modifications based on specific applications, whether in biomimetic studies, where polymersomes may be better-suited, or drug delivery, where microcapsules may be better-suited. The ability to fine-tune the properties of each protocell, including size, membrane or shell permeability, and surface morphology, highlights the potential for these methods to facilitate controlled and high-throughput generation of artificial cells with customized properties and functionalities.

## Conclusion

This study presents innovative methods for preparing artificial cells made of polymers, including polymersomes, protein-integrated polymersomes, and polymeric microcapsules, all compatible with a glass-capillary microfluidic device setup. Diverse polymersomes with uniform membranes are generated with the assistance of Pluronic F-68 in the outer phase, which adjusts the interfacial energies of double emulsion drops to induce spontaneous and complete dewetting. Moreover, multiple recombinant proteins are incorporated into these emulsion-templated polymer membranes while retaining their activity. Lastly, hollow PLGA microcapsules the size of biological cells, and with micron-sized surface features that resemble those found on the surface of natural cells, are prepared. These features are obtained by incorporating nanoparticles into the middle phase of the emulsion templates. The integration of these novel techniques not only simplifies the protocell production process but also broadens its potential applications, making it a valuable tool for both research and practical applications in multiple fields. The adaptability and efficiency of our method underscore its potential as a cornerstone technique in the advancing landscape of artificial cell fabrication by microfluidics.

### Supplementary Information

Below is the link to the electronic supplementary material.

Supplementary file 1 (pdf 827 KB)

Supplementary file 2 (avi 1450 KB)

Supplementary file 3 (avi 2279 KB)

Supplementary file 4 (avi 14179 KB)

Supplementary file 5 (avi 8850 KB)

## Data Availability

This manuscript has associated data in a data repository. [Authors’ comment: The authors declare that the data supporting the findings of this study are available within the paper. Should any raw data files be needed in another format they are available from the corresponding author upon reasonable request].
