# Supplementary material for: A microfluidic platform for the synthesis of polymer and polymer-protein-based protocells
Source: Eur Phys J E Soft Matter. 2024 Jun 3;47(6):37. doi: 10.1140/epje/s10189-024-00428-5 (PMC11147907; doi:10.1140/epje/s10189-024-00428-5)
Supplement: Supplementary file 1 — (pdf 827 KB) [file 10189_2024_428_MOESM1_ESM.pdf]

Supplementary Information for “A Microfluidic Platform for the Synthesis of Polymer and Polymer-Protein-Based Protocells”

Jessica Ann O’Callaghan[1], Neha P Kamat[2†], Kevin B Vargo[1†], Rajarshi Chattaraj[1†], Daeyeon Lee[1*], Daniel A. Hammer[1,2*]

[1]Department of Chemical and Biomolecular Engineering, University of Pennsylvania, 210 S 33rd Street, Philadelphia, 19104, Pennsylvania, United States.

[2]Department of Biongineering, University of Pennsylvania, 210 S 33rd Street, Philadelphia, 19104, Pennsylvania, United States.

*Corresponding author(s). E-mail(s): daeyeon@seas.upenn.edu; hammer@seas.upenn.edu;

†These authors contributed equally to this work.

1 Interfacial Tension Measurements

1.1 Interfacial Tensions Between I-M and M-O Phases

The interfacial tensions between the inner and middle (I-M) and middle and outer (M-O) phases are determined by an inverted pendant drop method using an Attention Theta (OneAttention) tensiometer. The interfacial tension value is recorded until the pendant drop detaches from the dispenser. Although interfacial tension measurements for the I-M and M-O without F-68 samples are recorded for >5 min, the interfacial tension rapidly decreases in the M-O with F-68 sample for ≈1 min before the drop detaches from the dispensing needle. To systematize our reported measurements, a time period of 1 min is chosen (Figure S2).

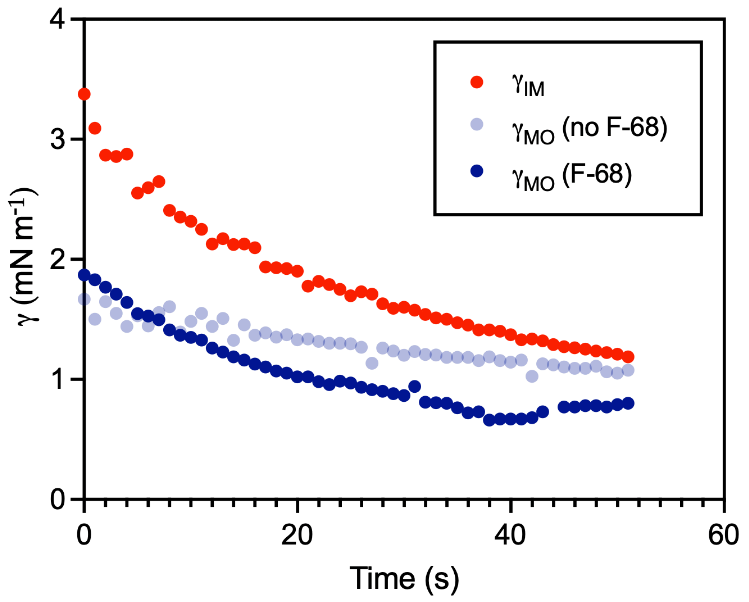

Figure S1 The surface tension over time between I-M and M-O, with and without F-68. Each interfacial tension measurement is taken three times and averaged.

1.2 Interfacial Tension Between I-O Phases

Since directly measuring the interfacial tension of the bilayer membrane between two water phases is not feasible, the method described involves estimating the interfacial tension of a monolayer that forms a bilayer between two water droplets (I and O) in an oil phase (M)[1–3]. This is achieved by conducting adhesion experiments with two polymer-stabilized water drops dispersed in a continuous oil phase. The interfacial tensions are calculated based on the balance of forces in the system.

The primary formula used in this process is the Neumann triangle equation:

$$\gamma_{IO} = -(\gamma_{IM} cos(\theta_1) + (\gamma_{MO} cos(\theta_2)) \tag{S1}$$

where γ_{IO} represents the interfacial tension of the monolayer forming the bilayer, and γ_{IM} and γ_{MO} are the interfacial tensions between the water and oil phases. The angles, θ_1 and θ_2, are the angles formed at the point of contact between the water droplets and the oil phase. Essentially, θ_1 corresponds to the angle at the interface between the oil phase and water droplet I, and θ_2 corresponds to the angle at the interface between the oil phase and water droplet O. These angles range between $\pi/2$ and π.

The adhesion energy (ΔF) of the system is given by the following equation:

$$\Delta F = \gamma_{IM} + \gamma_{MO} - \gamma_{IO} \tag{S2}$$

The interfacial tensions between the oil-water phases (γ_{IM} and γ_{MO}) are determined using the pendant drop method, and γ_{IM} is calculated using Equation S1. The method also considers two boundary conditions based on the behavior of the droplets:

Case 1: When the angles θ_1 and θ_2 equal π, there is no adhesion between the drops, leading to $\gamma_{IO} = \gamma_{IM} + \gamma_{MO}$ and $\Delta F = 0$. This indicates the absence of an adhesion force, although the possibility of γ_{IO} being greater than $\gamma_{IM} + \gamma_{MO}$ ($\Delta F < 0$) is also possible. For simplicity of the calculation, we only consider $\gamma_{IO} = \gamma_{IM} + \gamma_{MO}$ for Case 1.

Case 2: When the angles θ_1 and θ_2 are $\pi/2$, the doublet forms a spherical shape. In this scenario, $\gamma_{IO} = 0$, and $\Delta F = \gamma_{IM} + \gamma_{MO} > 0$, suggesting that polymersomes composed of this bilayer would have a zero interfacial tension.

For the adhesion experiment, I and O drops are independently formed at the ends of two separate 20 μm capillaries submerged in M (2 mg/mL PEO_{30}-b-PBD_{46} in 30:70 chloroform:hexane). These drops are brought into contact and their configurations are recorded (Videos S3 and S4) on an optical microscope (Nikon Diaphot 300) for subsequent analysis.

2 Avidin Mixed with Oleosin-Blended Polymersomes

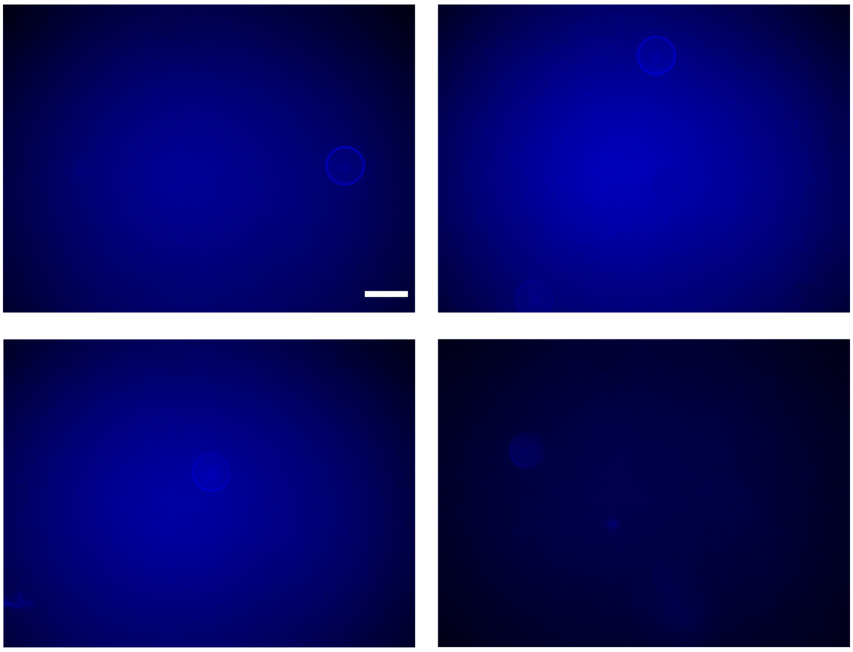

Figure S2 Fluorescent images of avidin (Alexa Fluor^{TM} 488 conjugate) incubated with PEO_{30}-b-PBD_{46} polymersomes prepared with biotin-functionalized 25-30G-30(-) (scale bar = 100 μm). The signal at the polymersome edge is higher than the background for 8 out of 9 vesicles imaged.

Video S1 - Dewetting of W/O/W double droplets to obtain polymersomes, monitored with optical microscopy (scale bar = 100 μm)

Video S2 - Dewetting of W/O/W droplets monitored with fluorescence microscopy (scale bar = 100 μm)

Video S3 - Drop adhesion experiment between inner and outer phases submerged in middle phase without F-68, showing no adhesion (scale bar = 20 μm)

Video S4 - Inner and outer drops submerged in middle phase with F-68, which adhere together (scale bar = 20 μm)

References

[1] Deng, N.-N., Yelleswarapu, M., Huck, W.T.: Monodisperse uni-and multicompartment liposomes. Journal of the American Chemical Society **138**(24), 7584–7591 (2016)

[2] Torza, S., Mason, S.: Coalescence of two immiscible liquid drops. Science **163**(3869), 813–814 (1969)

[3] Thiam, A.R., Bremond, N., Bibette, J.: Adhesive emulsion bilayers under an electric field: from unzipping to fusion. Physical review letters **107**(6), 068301 (2011)
